# The 150 most important questions in cancer research and clinical oncology series: questions 94–101: *Edited by Cancer Communications*

**DOI:** 10.1186/s40880-018-0341-9

**Published:** 2018-11-26

**Authors:** Cancer Communications

**Affiliations:** Sun Yat-sen University Cancer Center, Guangzhou, 510060 P. R. China

**Keywords:** Tumor origin, Polyploid giant cancer cell, Pancreatic ductal adenocarcinoma, Biomarker, Liquid biopsy, Spontaneous animal model, Metastasis, Chemotherapy, Immunotherapy, Precision treatment, Vaccine immunization, Metformin, Circulating tumor cell, Circulating tumor DNA, CpG methylation, Methylation haplotype block, Phytochemicals, P-Glycoprotein, Multi-drug resistance, P-Glycoprotein inhibitor, Epithelial-to-mesenchymal transition, Migration, Pro-migratory gene

## Abstract

Since the beginning of 2017, *Cancer Communications* (former title: *Chinese Journal of Cancer*) has published a series of important questions regarding cancer research and clinical oncology, to provide an enhanced stimulus for cancer research, and to accelerate collaborations between institutions and investigators. In this edition, the following 8 valuable questions are presented. Question 94. The origin of tumors: time for a new paradigm? Question 95. How can we accelerate the identification of biomarkers for the early detection of pancreatic ductal adenocarcinoma? Question 96. Can we improve the treatment outcomes of metastatic pancreatic ductal adenocarcinoma through precision medicine guided by a combination of the genetic and proteomic information of the tumor? Question 97. What are the parameters that determine a competent immune system that gives a complete response to cancers after immune induction? Question 98. Is high local concentration of metformin essential for its anti-cancer activity? Question 99. How can we monitor the emergence of cancer cells anywhere in the body through plasma testing? Question 100. Can phytochemicals be more specific and efficient at targeting P-glycoproteins to overcome multi-drug resistance in cancer cells? Question 101. Is cell migration a selectable trait in the natural evolution of carcinoma?

## Text

Until now, the battle against cancer is still ongoing, but there are also ongoing discoveries being made. Milestones in cancer research and treatments are being achieved every year; at a quicker pace, as compared to decades ago. Likewise, some cancers that were considered incurable are now partly curable, lives that could not be saved are now being saved, and for those with yet little options, they are now having best-supporting care. With an objective to promote worldwide cancer research and even accelerate inter-countries collaborations, since the beginning of 2017, *Cancer Communications* (former title: *Chinese Journal of Cancer*) has launched a program of publishing 150 most important questions in cancer research and clinical oncology [1]. We are providing a platform for researchers to freely voice-out their novel ideas, and propositions to enhance the communications on how and where our focus should be placed [2–13]. In this edition, 8 valuable and inspiring questions, Question 94–101, from highly distinguished professionals from different parts of the world are presented. If you have any novel proposition(s) and Question(s), please feel free to contact Ms. Ji Ruan via email: ruanji@sysucc.org.cn.

## Question 94: The origin of tumors: time for a new paradigm?

### Background and implications

*“There is no worse blind man than the one who doesn’t want to see. There is no worse deaf man than the one who doesn’t want to hear. And there is no worse madman than the one who doesn’t want to understand.”*—Ancient Proverb

In the past half-century, cancer biologists have focused on a dogma in which cancer was viewed as a proliferative disease due to mechanisms that activate genes (oncogenes) to promote cell proliferation or inactivate genes (tumor suppressor genes) to suppress tumor growth. In retrospect, these concepts were established based on functional selections, by using tissue culture (largely mouse NIH 3T3 cells) for the selection of transformed foci at the time when we knew virtually nothing about the human genome [14]. However, it is very difficult to use these genes individually or in combinations to transform primary human cells. Further, the simplified view of uncontrolled proliferation cannot explain the tumor as being a malignant organ or a teratoma, as observed by pathologists over centuries. Recently, the cancer genomic atlas project has revealed a wide variety of genetic alterations ranging from no mutation to multiple chromosomal deletions or fragmentations, which make the identification of cancer driver mutations very challenging in a background of such a massive genomic rearrangement. Paradoxically, this increase the evidences demonstrating that the oncogenic mutations are commonly found in many normal tissues, further challenging the dogma that genetic alteration is the primary driver of this disease.

**Fig. 1:**
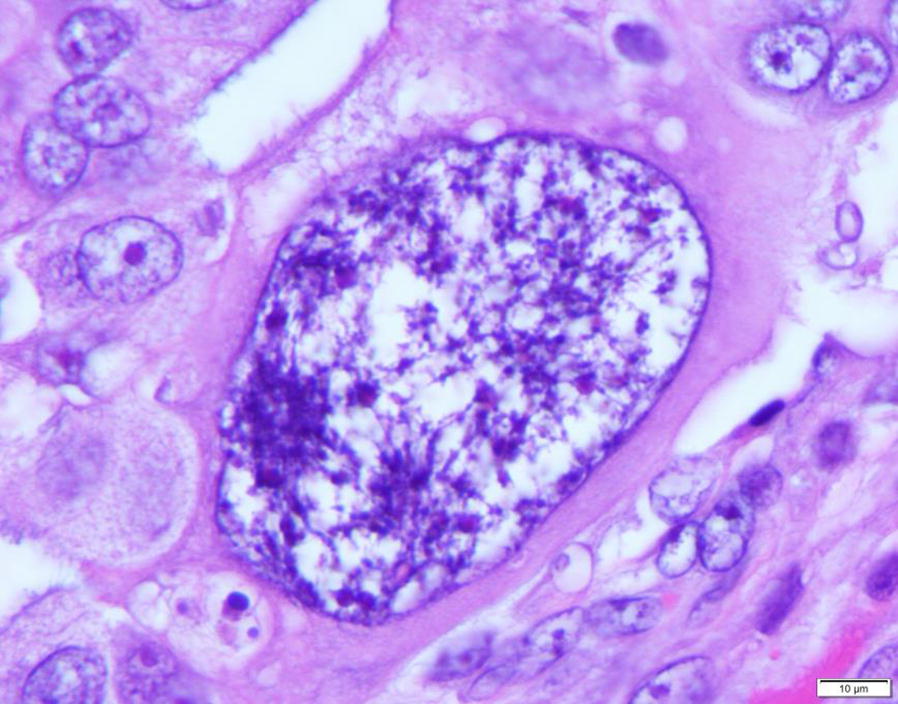
One mononucleated polyploid giant cancer cell (PGCC) in the background of regular size diploid cancer cells. The PGCC can be seen to be at least 100 times larger than that of regular cancer cells

Logically, the birth of a tumor should undergo an embryonic-like development at the beginning, similar to that of a human. However, the nature of such somatic-derived early embryo has been elusive. Recently, we provided evidence to show that polyploid giant cancer cells (PGCCs), which have been previously considered non-dividing, are actually capable of self-renewal, generating viable daughter cells via amitotic budding, splitting and burst, and capable of acquisition of embryonic-like stemness [15–17]. The mode of PGCC division is remarkably similar to that of blastomere, a first step in human embryogenesis following fertilization. The blastomere nucleus continuously divides 4–5 times without cytoplasmic division to generate 16–32 cells and then to form compaction/morulae before developing into a blastocyst [18]. Based on these data and similarity to the earliest stage of human embryogenesis, I propose a new theory that tumor initiation can be achieved via a dualistic origin, similar to the first step of human embryogenesis via the formation of blastomere-like cells, i.e. the activation of blastomere or blastomere-like cells which leads to the dedifferentiation of germ cells or somatic cells, respectively, which is then followed by the differentiation to generate their respective stem cells, and the differentiation arrest at a specific developmental hierarchy leading to tumor initiation [19]. The somatic-derived blastomere-like cancer stem cell follows its own mode of cell growth and division and is named as the giant cell cycle. This cycle includes four distinct but overlapping phases: the initiation, self-renewal, termination, and stability phases. The giant cell cycle can be tracked in vitro and in vivo due to their salient giant cell morphology (Fig. 1).

This new theory challenges the traditional paradigm that cancer is a proliferative disease, and proposes that the initiation of cancer requires blastomere-like division that is similar to that of humans before achieving stable proliferation at specific developmental hierarchy in at least half of all human cancers. This question calls for all investigators in the cancer research community to investigate the role of PGCCs in the initiation, progression, resistance, and metastasis of cancer and to look for novel agents to block the different stages of the giant cell cycle.

The histopathology (phenotype) of cancers has been there all the time. It is just the theory of cancer origin proposed by scientists that changes from time to time. After all, trillions of dollars have been invested in fighting this disease by basing on its genetic origin in the past half-century, yet, little insight has been gained [14]. Here are two quotes from Einstein: “Insanity: doing the same thing over and over again expecting different results”, and “We cannot solve our problems with the same thinking we used when created them”.

In short, it is time to change our mindset and to start pursuing PGCCs, which we can observe under the microscope. But with very little understanding about these cells, it is time for a shift in paradigm.

#### Submitter

Jinsong Liu.

#### Affiliation

Department of Pathology, The University of Texas MD Anderson Cancer Center, Houston, TX 77030-4095, USA.

#### Email address

jliu@mdanderson.org

## Question 95: How can we accelerate the identification of biomarkers for the early detection of pancreatic ductal adenocarcinoma?

### Background and implications

Pancreatic ductal adenocarcinoma (PDAC) is one of the most lethal cancers in the world with a dismal 5-year overall survival rate of less than 5%; which has not been significantly improved since the past decades. Although surgical resection is the only option for curative treatment of PDAC, only 15%–20% of patients with PDAC have the chance to undergo curative resection, leaving the rest with only palliative options in hope for increasing their quality of life; since they were already at unresectable and non-curative stages at their first diagnosis.

The lack of specific symptoms in the early-stage of PDAC is responsible for rendering an early diagnosis difficult. Therefore, more sensitive and specific screening methodologies for its early detection is urgently needed to improve its diagnosis, starting early treatments, and ameliorating prognoses. The diagnosis so far relies on imaging modalities such as abdominal ultrasound, computed tomography (CT), magnetic resonance imaging (MRI), endoscopic ultrasound (EUS), endoscopic retrograde cholangiopancreatography (ERCP), and positron emission tomography (PET). One may propose to screen for pancreatic cancer in high-risk populations, which is highly recommended, however screening intervention for all the people is not a wise choice; when considering the relatively low prevalence of PDAC, and the difficulty for diagnosing it in its early stage [20].

Therefore, alternative diagnostic tools for early detection of PDAC are highly expected. Among the biomarkers currently used in clinical practice, carbohydrate antigen 19–9 (CA19–9) is among the most useful one for supporting the diagnosis of PDAC, but it is neither sufficiently sensitive nor specific for its early detection. Yachida et al. reported in 2010 that the initiating mutation in the pancreas occurs approximately two decades before the PDAC to start growing in distant organs [21], which indicates a broad time of the window of opportunity for the early detection of PDAC. With the advancement in next-generation sequencing technology, the number of reported studies regarding novel potential molecular biomarkers in bodily fluids including the blood, feces, urine, saliva, and pancreatic juice for early detection of PDAC has been increasing. Such biomarkers may be susceptible to detect mutations at the genetic or epigenetic level, identifying important non-coding RNA (especially microRNA and long non-coding RNA), providing insights regarding the metabolic profiles, estimating the tumor level in liquid biopsies (circulating free DNA, circulating tumor cells and exosomes), and so on.

Another approach to identifying biomarkers for the early detection of pancreatic cancer is using animal models. In spontaneous animal models of pancreatic cancer, such as Kras-mutated mouse models, it is expected that by high throughput analyses of the genetic/epigenetic/proteomic alterations, some novel biomarkers might be able to be identified. For instance, Sharma et al. reported in 2017 that the detection of phosphatidylserine-positive exosomes enabled the diagnosis of early-stage malignancies in LSL-Kras^G12D^, Cdkn2a^lox/lox^: p48^Cre^ and LSL-Kras^G12d/+^, LSL-Trp^R172H/+^, and P48^Cre^ mice [22].

These analyses in clinical samples or animal models hold the clues for the early detection of PDAC, however, further studies are required to validate their diagnostic performance. What’s most important, will be the lining-up of these identified prospective biomarkers, to validate their sensitivities and specificities. This will determine their potential for widespread clinical applicability, and hopefully, accelerate the early diagnosis of PDAC.

#### Submitter

Mikiya Takao^1,2^, Hirotaka Matsuo^2^, Junji Yamamoto^1^, and Nariyoshi Shinomiya^2^.

#### Affiliation

^1^Department of Surgery, National Defense Medical College, 3-2 Namiki, Tokorozawa, Saitama 359-8513, Japan; ^2^Department of Integrative Physiology and Bio-Nano Medicine, National Defense Medical College, 3-2 Namiki, Tokorozawa, Saitama 359-8513, Japan.

#### E-mail address

mitakao-tky@umin.ac.jp; hmatsuo@ndmc.ac.jp; jyamamot@ndmc.ac.jp; shinomi@ndmc.ac.jp

## Question 96: Can we improve the treatment outcomes of metastatic pancreatic ductal adenocarcinoma through precision medicine guided by a combination of the genetic and proteomic information of the tumor?

### Background and implications

Pancreatic ductal adenocarcinoma (PDAC) is one of the most malignant cancers, and nearly half of the patients had metastatic PDAC when they are initially diagnosed. When they are accompanied by metastatic tumors, unlike most solid cancer, PDAC cannot be cured with primary surgical resection alone [23, 24]. Also, since PDAC has poor responses to conventional therapies, improvements in adjunctive treatment approach including chemo- and immuno-therapy are earnestly required. From this standpoint, recent results regarding the differences in the molecular evolution of pancreatic cancer subtypes provide a new insight into its therapeutic development [25], which may lead to the improvement of the prognosis of not only metastatic PDAC but also of locally advanced or recurrent PDAC.

In fact, new chemotherapeutic regimens such as the combination of gemcitabine with nab-paclitaxel and FOLFIRINOX have been reported to show improved prognosis despite a lack of examples of past successes in the treatment of patients with metastatic PDAC who had undergone R0 resection [26]. While many mutations including *KRAS*, *CDKN2A*, *TP53,* and *SMAD4* are associated with pancreatic carcinogenesis, no effective molecular targeted drug has been introduced in the clinical setting so far. A recent report of a phase I/II study on refametinib, a MEK inhibitor, indicated that *KRAS* mutation status might affect the overall response rate, disease control rate, progression-free survival, and overall survival of PDAC in combination with gemcitabine [27].

While immunotherapy is expected to bring a great improvement in cancer treatment, until now, immune checkpoint inhibitors have achieved limited clinical benefit for patients with PDAC. This might be because PDAC creates a uniquely immunosuppressive tumor microenvironment, where tumor-associated immunosuppressive cells and accompanying desmoplastic stroma prevent the tumor cells from T cell infiltration. Recently reported studies have indicated that immunotherapy might be effective when combined with focal adhesion kinase (FAK) inhibitor [28] or IL-6 inhibitor [29], but more studies are required to validate their use in clinical practice.

As such, we believe that if the dynamic monitoring of drug sensitivity/resistance in the individual patients is coupled with precision treatment based on individualized genetics/epigenetics/proteomics alterations in the patients’ tumor, this could improve the treatment outcomes of PDAC.

#### Submitter

Mikiya Takao^1,2^, Hirotaka Matsuo^2^, Junji Yamamoto^1^, and Nariyoshi Shinomiya^2.^

#### Affiliation

^1^Department of Surgery, National Defense Medical College, 3-2 Namiki, Tokorozawa, Saitama 359-8513, Japan; ^2^Department of Integrative Physiology and Bio-Nano Medicine, National Defense Medical College, 3-2 Namiki, Tokorozawa, Saitama 359-8513, Japan.

#### E-mail address

mitakao-tky@umin.ac.jp; hmatsuo@ndmc.ac.jp; jyamamot@ndmc.ac.jp; shinomi@ndmc.ac.jp

## Question 97: What are the parameters that determine a competent immune system that gives a complete response to cancers after immune induction?

### Background and implications

Recently, cancer immunotherapy has shown great clinical benefit in multiple types of cancers [30–32]. It has provided new approaches for cancer treatment. However, it has been observed that only a fraction of patients respond to immunotherapy.

Much effort has been made to identify markers for immunotherapeutic response. Tumor mutation burden (TMB), mismatch repair (MMR) deficiency, PD-L1 expression, and tumor infiltration lymphocyte (TIL) have been found to be associated with an increased response rate in checkpoint blockade therapies. Unfortunately, a precise prediction is still challenging in this field. Moreover, when to stop the treatment of immunotherapy is an urgent question that remains to be elucidated.

In other words, there is no available approach to determine if a patient has generated a good immune response against the cancer after immunotherapy treatments. All of these indicate the complexity and challenges that reside for implementing novel man-induced cancer-effective immune response therapeutics. A variety of immune cells play collaborative roles at different stages to recognize antigens and eventually to generate an effective anti-cancer immune response. Given the high complexity of the immune system, a rational evaluation approach is needed to cover the whole process. Moreover, we need to perfect vaccine immunization and/or in vitro activation of T cells to augment the function of the immune system; particularly the formation of immune memory.

#### Submitter

Edison Liu^1^, Penghui Zhou^2^, Jiang Li^2^.

#### Affiliation

^1^The Jackson Laboratory, Bar Harbor, ME 04609, USA; ^2^Sun Yat-sen University Cancer Center, Guangzhou, Guangdong 510060, P. R. China.

#### Email address

Ed.Liu@jax.org; zhouph@sysucc.org.cn; lijiang@sysucc.org.cn

## Question 98: Is high local concentration of metformin essential for its anti-cancer activity?

### Background and implications

Metformin was approved as a first line of anti-diabetic drug since decades. Interestingly, the fact that clinical epidemiological studies have shown that metformin can reduce the risk of a variety of cancers stimulates considerable recognition to explore its anticancer activity.

Although the in vitro and in vivo experimental results have demonstrated that metformin can have some potential anti-tumor effects, more than 100 clinical trials did not achieve such desirable results [33]. We and others believe that the main problem resides in the prescribing doses used. For cancer treatment, a much higher dose may be needed for observing any anti-tumor activities, as compared to the doses prescribed for diabetics [34–36].

Further, if the traditional local/oral administration approach is favored, the prescribed metformin may not be at the required dose-concentration once it reaches the blood to have the effective anti-cancer activities. We, therefore, propose that intravesical instillation of metformin into the bladder lumen could be a promising way to treat for bladder cancer, at least. We have already obtained encouraging results both in vitro and in vivo experiments, including in an orthotopical bladder cancer model [36, 37]. Now, we are waiting to observe its prospective clinical outcome.

#### Submitter

Mei Peng^1^, Xiaoping Yang^2^.

#### Affiliation

^1^Department of Pharmacy, Xiangya Hospital, Central South University. Changsha, Hunan 410083, P. R. China; ^2^Key Laboratory of Study and Discovery of Small Targeted Molecules of Hunan Province, Department of Pharmacy, School of Medicine, Hunan Normal University, Changsha, Hunan 410013, P. R. China.

#### Email address

Meipeng@csu.edu.cn; Xiaoping.Yang@hunnu.edu.cn

## Question 99: How can we monitor the emergence of cancer cells anywhere in the body through plasma testing?

### Background and implications

The early detection of cancer is still a relentless worldwide challenge. The sensitivity and specificity of traditional blood tumor markers and imaging technologies are still to be greatly improved. Hence, novel approaches for the early detection of cancer are urgently needed.

The emergence of liquid biopsy technologies opens a new driveway for solving such issues. According to the definition of the National Cancer Institute of the United States, a liquid biopsy is a test done on a sample of blood to look for tumorigenic cancer cells or pieces of tumor cells’ DNA that are circulating in the blood [38]. This definition implies two main types of the current liquid biopsy: one that detects circulating tumor cells and the other that detects non-cellular material in the blood, including tumor DNA, RNA, and exosomes.

Circulating tumor cells (CTCs) are referred to as tumor cells that have been shed from the primary tumor location and have found their way to the peripheral blood. CTCs were first described in 1869 by an Australian pathologist, Thomas Ashworth, in a patient with metastatic cancer [39]. The importance of CTCs in modern cancer research began in the mid-1990s with the demonstration that CTCs exist early in the course of the disease.

It is estimated that there are about 1–10 CTCs per mL in whole blood of patients with metastatic cancer, even fewer in patients with early-stage cancer [40]. For comparison, 1 mL of blood contains a few million white blood cells and a billion erythrocytes. The identification of CTCs, being in such low frequency, requires some special tumoral markers (e.g., EpCAM and cytokeratins) to capture and isolate them. Unfortunately, the common markers for recognizing the majority of CTCs are not effective enough for clinical application [41]. Although accumulated evidences have shown that the presence of CTCs is a strong negative prognostic factor in the patients with metastatic breast, lung and colorectal cancers, detecting CTCs might not be an ideal branch to hold on for the hope of early cancer detection [42–45].

Circulating tumor DNA (ctDNA) is tumor-derived fragmented DNA in the circulatory system, which is mainly derived from the tumor cell death through necrosis and/or apoptosis [46]. Given its origin, ctDNA inherently carries cancer-specific genetic and epigenetic aberrations, which can be used as a surrogate source of tumor DNA for cancer diagnosis and prognostic prediction. Ideally, as a noninvasive tumor early screening tool, a liquid biopsy test should be able to detect many types of cancers and provide the information of tumor origin for further specific clinical management. In fact, the somatic mutations of ctDNA in different types of tumor are highly variable, even in the different individuals with the same type of tumor [47]. Additionally, most tumors do not possess driver mutations, with some notable exceptions, which make the somatic mutations of ctDNA not suitable for early detection of the tumor.

Increased methylation of the promoter regions of tumor suppressor genes is an early event in many types of tumor, suggesting that altered ctDNA methylation patterns could be one of the first detectable neoplastic changes associated with tumorigenesis [48]. ctDNA methylation profiling provides several advantages over somatic mutation analysis for cancer detection including higher clinical sensitivity and dynamic range, multiple detectable methylation target regions, and multiple altered CpG sites within each targeted genomic region. Further, each methylation marker is present in both cancer tissue and ctDNA, whereas only a fraction of mutations present in cancer tissue could be detected in ctDNA.

In 2017, there were two inspiring studies that revealed the values of using ctDNA methylation analysis for cancer early diagnosis [49, 50]. After partitioning the human genome into blocks of tightly coupled CpG methylation sites, namely methylation haplotype blocks (MHBs), Guo and colleagues performed tissue-specific methylation analyses at the MHBs level to accurately determine the tissue origin of the cancer using ctDNA from their enrolled patients [49]. In another study, Xu and colleagues identified a hepatocellular carcinoma (HCC) enriched methylation marker panel by comparing the HCC tissue and blood leukocytes from normal individuals and showed that methylation profiles of HCC tumor DNA and matched plasma ctDNA were highly correlated. In this study, after quantitative measurement of the methylation level of candidate markers in ctDNA from a large cohort of 1098 HCC patients and 835 normal controls, ten methylation markers were selected to construct a diagnostic prediction model. The proposed model demonstrated a high diagnostic specificity and sensitivity, and was highly correlated with tumor burden, treatment response, and tumor stage [50].

With the rapid development of highly sensitive detection methods, especially the technologies of massively parallel sequencing or next-generation sequencing (NGS)-based assays and digital PCR (dPCR), we strongly believe that the identification of a broader “pan-cancer” methylation panel applied for ctDNA analyses, probably in combination with detections of somatic mutation and tumor-derived exosomes, would allow more effective screening for common cancers in the near future.

#### Submitter

Edison Liu^1^, Hui-Yan Luo^2^.

#### Affiliation

^1^The Jackson Laboratory, Bar Harbor, ME 04609, USA; ^2^Sun Yat-sen University Cancer Center, Guangzhou, Guangdong 510060, P. R. China.

#### Email address

Ed.Liu@jax.org; luohy@sysucc.org.cn

## Question 100: Can phytochemicals be more specific and efficient at targeting P-glycoproteins to overcome multi-drug resistance in cancer cells?

### Background and implications

Though several anticancer agents are approved to treat different types of cancers, their full potentials have been limited due to the occurrence of drug resistance. Resistance to anticancer drugs develops by a variety of mechanisms, one of which is increased drug efflux by transporters. The ATP-binding cassette (ABC) family drug efflux transporter P-glycoprotein (P-gp or multi-drug resistance protein 1 [MDRP1]) has been extensively studied and is known to play a major role in the development of multi-drug resistance (MDR) to chemotherapy [51]. In brief, overexpressed P-gp efflux out a wide variety of anticancer agents (e.g.: vinca alkaloids, doxorubicin, paclitaxel, etc.), leading to a lower concentration of these drugs inside cancer cells, thereby resulting in MDR. Over the past three decades, researchers have developed several synthetic P-gp inhibitors to block the efflux of anticancer drugs and have tested them in clinical trials, in combination with chemotherapeutic drugs. But none were found to be suitable enough in overcoming MDR and to be released for marketing, mainly due to the side effects associated with cross-reactivity towards other ABC transporters (BCRP and MRP-1) and the inhibition of CYP450 drug metabolizing enzymes [52, 53].

On the other hand, a number of phytochemicals have been reported to have P-gp inhibitory activity. Moreover, detailed structure–activity studies on these phytochemicals have delineated the functional groups essential for P-gp inhibition [53, 54]. Currently, one of the phytochemicals, tetrandrine (CBT-1^®^; NSC-77037), is being used in a Phase I clinical trial (http://www.ClinicalTrials.gov; NCT03002805) in combination with doxorubicin for the treatment of metastatic sarcoma. Before developing phytochemicals or their derivatives as P-gp inhibitors, they need to be investigated thoroughly for their cross-reactivity towards other ABC transporters and CYP450 inhibition, in order to avoid toxicities similar to the older generation P-gp inhibitors that have failed in clinical trials.

Therefore, the selectivity for P-gp over other drug transporters and drug metabolizing enzymes should be considered as important criterias for the development of phytochemicals and their derivatives for overcoming MDR.

#### Submitter

Mohane Selvaraj Coumar and Safiulla Basha Syed.

#### Affiliation

Centre for Bioinformatics, School of Life Sciences, Pondicherry University, Kalapet, Puducherry 605014, India.

#### Email address

mohane@bicpu.edu.in; safi742@gmail.com

## Question 101: Is cell migration a selectable trait in the natural evolution of carcinoma?

The propensity of solid tumor malignancy to metastasize remains the main cause of cancer-related death, an extraordinary unmet clinical need, and an unanswered question in basic cancer research. While dissemination has been traditionally viewed as a late process in the progression of malignant tumors, amount of evidence indicates that it can occur early in the natural history of cancer, frequently when the primary lesion is still barely detectable.

A prerequisite for cancer dissemination is the acquisition of migratory/invasive properties. However, whether, and if so, how the migratory phenotype is selected for during the natural evolution of cancer and what advantage, if any, it may provide to the growing malignant cells remains an open issue. The answers to these questions are relevant not only for our understating of cancer biology but also for the strategies we adopt in an attempt of curbing this disease. Frequently, indeed, particularly in pharmaceutical settings, targeting migration has been considered much like trying “to shut the stable door after the horse has bolted” and no serious efforts in pursuing this aim has been done.

We argue, instead, that migration might be an intrinsic cancer trait that much like proliferation or increased survival confers to the growing tumor masses with striking selective advantages. The most compelling evidence in support for this contention stems from studies using mathematical modeling of cancer evolution. Surprisingly, these works highlighted the notion that cell migration is an intrinsic, selectable property of malignant cells, so intimately intertwined with more obvious evolutionarily-driven cancer traits to directly impact not only on the potential of malignant cells to disseminate but also on their growth dynamics, and ultimately provide a selective evolutionary advantage. Whether in real life this holds true remains to be assessed, nevertheless, work of this kind defines a framework where the acquisition of migration can be understood in a term of not just as a way to spread, but also to trigger the emergence of malignant clones with favorable genetic or epigenetic traits.

Alternatively, migratory phenotypes might emerge as a response to unfavorable conditions, including the mechanically challenging environment which tumors, and particularly epithelial-derived carcinoma, invariably experience. Becoming motile, however, may not per se being fixed as phenotypic advantageous traits unless it is accompanied or is causing the emergence of specific traits, including drug resistance, self-renewal, and survival. This might be the case, for example, during the process of epithelial-to-mesenchymal transition (EMT), which is emerging as an overarching mechanism for dissemination. EMT, indeed, may transiently equip individual cancer cells not only with migratory/invasive capacity but also with increased resistance to drug treatment, stemness potential at the expanse of fast proliferation.

Thus, within this framework targeting pro-migratory genes, proteins and processes may become a therapeutically valid alternative or a complementary strategy not only to control carcinoma dissemination but also its progression and development.

### Submitter

Giorgio Scita.

### Affiliation

IFOM, The FIRC Institute of Molecular Oncology, Via Adamello 16, 20139 Milan, Italy; Department of Oncology and Hemato-Oncology (DIPO), School of Medicine, University of Milan, Via Festa del Perdono 7, 20122, Italy.

### Email address

giorgio.scita@ifom.eu
